# Understanding Technology Anxiety Among Older Adults with Chronic Diseases: The Roles of Perceived Ease of Use and Perceived Usefulness

**DOI:** 10.3390/healthcare14193160

**Published:** 2026-09-23

**Authors:** Ting Chen, Meiling Li, Didi Sun, Renlan Gao, Qing Hong, Xiaoqing Lv

**Affiliations:** School of Nursing, Anhui Medical University, Hefei 231200, China; 2445011338@stu.ahmu.edu.cn (T.C.); 2445011204@stu.ahmu.edu.cn (M.L.); 2445011234@stu.ahmu.edu.cn (D.S.); 2445011276@stu.ahmu.edu.cn (R.G.); 2445011279@stu.ahmu.edu.cn (Q.H.)

**Keywords:** technology anxiety, older adults with chronic diseases, eHealth literacy, chronic disease management self-efficacy, social support, technology acceptance model

## Abstract

**Highlights:**

**What are the main findings?**

- Older adults with chronic diseases reported measurable levels of technology anxiety, which were associated with individual and social factors.
- Perceived ease of use and perceived usefulness showed indirect associations with technology anxiety.

**What are the implications of the main findings?**

- Support strategies for older adults using digital health technologies may consider perceived ease of use and perceived usefulness.
- Understanding digital health perceptions may provide insights into technology anxiety.

**Abstract:**

**Purpose:** To examine the associations of individual-level factors (eHealth literacy, chronic disease management self-efficacy, and coping style) and social-level factors (social support) with technology anxiety among older adults with chronic diseases, and to explore the potential indirect associations involving perceived ease of use and perceived usefulness. **Method:** A cross-sectional observational study was conducted among older adults with chronic diseases, in which 470 participants were surveyed and 456 valid responses were obtained. Measures included the eHealth Literacy Scale, Self-Efficacy for Managing Chronic Disease Scale, Simplified Coping Style Questionnaire, Social Support Rating Scale, Scale of Perceived Ease of Use, Perceived Usefulness Scale and Technology Anxiety Scale. Structural equation modelling was applied to examine the direct and indirect associations among individual- and social-level factors, technology perceptions, and technology anxiety. **Results:** Higher eHealth literacy, chronic disease management self-efficacy, positive coping, and social support were positively associated with perceived ease of use and perceived usefulness, whereas negative coping was negatively associated with both. Perceived ease of use and perceived usefulness were negatively associated with technology anxiety. eHealth literacy, chronic disease management self-efficacy, and positive coping were negatively associated with technology anxiety, whereas negative coping was positively associated; social support showed no significant direct association. All five antecedent factors showed significant indirect associations with technology anxiety through perceived ease of use, perceived usefulness, and the sequential pathway from perceived ease of use to perceived usefulness. **Conclusions:** Individual- and social-level factors were associated with technology anxiety among older adults with chronic diseases, with perceived ease of use and perceived usefulness involved in these indirect associations. These findings may provide insights for understanding technology anxiety and support future research on digital health adaptation.

## 1. Introduction

The global population is ageing rapidly, with the number of older adults living with chronic diseases continuing to increase. According to the World Health Organization (WHO), the population aged 60 years and over is projected to rise from 1 billion in 2020 to 1.4 billion by 2030 [1]. In China, approximately 81% of older adults have at least one chronic disease, representing nearly 180 million individuals and accounting for around 70% of the overall disease burden [2,3].

In recent years, healthcare services have undergone a rapid transition towards digitalisation and intelligent systems. The WHO Global Strategy on Digital Health 2020–2025 highlights the role of digital technologies in improving the accessibility and efficiency of healthcare, positioning digital health as a central component of global health system development [4]. Consequently, patient-facing digital healthcare technologies, such as online appointment systems, hospital self-service devices, and smartphone-based healthcare applications, are increasingly integrated into healthcare services [5,6]. Older adults with chronic diseases often experience declines in physiological functioning and psychological adaptability, which may hinder their acceptance and use of digital health technologies [7]. Owing to the long-term nature of chronic conditions, this population typically has substantial and complex healthcare needs [8]. Their healthcare-seeking processes frequently involve multiple stages, including appointment scheduling, diagnostic examinations, retrieval of medical reports and follow-up care [5]. As healthcare services become increasingly digitalised, many of these processes are now conducted through digital technologies, placing additional demands on individuals’ ability to use and adapt to digital healthcare systems [4]. Consequently, older adults with chronic diseases may experience particular difficulties when engaging with digital health technologies, which may be associated with higher levels of technology anxiety [9]. Technology anxiety is also relevant to healthcare delivery and nursing practice because it may make it more difficult for older adults with chronic diseases to use online appointment systems, obtain health information, complete follow-up procedures, and access other digital healthcare services that are increasingly involved in long-term disease management [10]. It may also be associated with feelings of frustration, helplessness, and marginalisation, as well as lower confidence in self-management and perceived control over health-related situations [11].

Technology anxiety originated from the concept of “computer anxiety”, which initially referred to feelings of fear and apprehension associated with computer use [12]. With the continued evolution of digital technologies, the concept has expanded beyond computers to encompass a broader range of digital tools. Technology anxiety is now regarded as a multidimensional psychological phenomenon characterised by low self-esteem, negative emotional responses, discomfort and maladaptive beliefs related to technology use [13]. Although digital technologies may improve efficiency in daily life and healthcare delivery, they may also generate feelings of pressure, isolation and confusion, thereby contributing to anxiety, tension and frustration among users. Within healthcare contexts, technology anxiety refers to a psychological response characterised by tension, fear and avoidance when patients interact with new healthcare technologies [14].

Technology anxiety has been reported among older adults with chronic diseases [15]. Previous studies have identified several factors associated with technology anxiety in this population, including eHealth literacy and self-efficacy at the individual level and social support at the social level [16,17,18]. The relationship between coping style and technology anxiety has not been examined directly; however, broader research on anxiety in older adults suggests that positive coping is associated with lower anxiety, whereas avoidance-oriented coping is associated with more negative emotional responses [19]. Despite these findings, it remains unclear how these factors are related to technology perceptions and technology anxiety when considered together, including their potential direct and indirect associations. The present study therefore focused on eHealth literacy, chronic disease management self-efficacy, coping style, and social support within an integrated theoretical framework. eHealth literacy reflects an individual’s ability to access, understand, and use electronic health information, self-efficacy reflects beliefs about one’s capability to manage health-related tasks, and coping style reflects cognitive and behavioural responses to stressful situations [20,21,22]. These constructs were therefore considered individual-level factors. In contrast, social support reflects interpersonal resources available from family members, friends, and other social contacts and was therefore considered a social-level factor [23].

To provide an integrated theoretical framework, the present study drew on the Technology Acceptance Model (TAM). Perceived ease of use (PEOU) and perceived usefulness (PU) are two core cognitive beliefs in TAM. PEOU refers to the degree to which a person believes that using a technology would require little effort, whereas PU refers to the degree to which a person believes that using a technology would enhance performance [24]. Within the original TAM, PEOU is theorised to be positively related to PU, such that technologies perceived as easier to use are also more likely to be perceived as useful [24]. PEOU and PU were therefore conceptualized as technology-specific cognitive evaluations, whereas technology anxiety was treated as a psychological response associated with these evaluations. This ordering is supported by research in older adults that has modelled perceived usefulness upstream of technology anxiety and by systematic-review evidence linking technology-related evaluations, including ease of use and usefulness, with digital health technology anxiety [16,25]. Together, these findings suggest that older adults’ perceptions of the ease and usefulness of digital health technologies may be related to their technology-related emotional responses. Although technology anxiety has often been conceptualised as an external antecedent of technology acceptance in previous TAM-based research, the present study addresses a different research question: why some older adults with chronic diseases experience greater technology anxiety when engaging with digital healthcare technologies. Technology anxiety was therefore positioned as the focal psychological outcome, whereas PEOU and PU were treated as cognitive evaluations potentially associated with this emotional response. Building on the original TAM, the present study further incorporated eHealth literacy, chronic disease management self-efficacy, coping style, and social support as antecedent factors (Figure 1). In this extended framework, the PEOU–PU relationship was derived from the original TAM, whereas the proposed associations of these antecedent factors with PEOU, PU, and technology anxiety represented extensions of the model. PEOU and PU were also examined as potential pathways through which indirect associations with technology anxiety might occur.

Previous evidence suggests that eHealth literacy is positively associated with PEOU and PU of digital health technologies among older adults [26]. Given that these perceptions are core cognitive evaluations in TAM, higher eHealth literacy may also be associated with lower technology anxiety, potentially through a better understanding of the functions, benefits, and use of digital health technologies. Empirical studies in chronic disease management contexts have also shown that self-efficacy is related to patients’ perceptions of digital health technologies [27]. For example, studies of computer-based chronic disease self-monitoring systems and smartphone-based chronic disease management technologies found that higher self-efficacy was associated with greater perceived ease of use, while perceived ease of use was positively related to perceived usefulness [28]. Older adults with stronger disease management self-efficacy may therefore be more likely to perceive digital health technologies as manageable and useful for self-management. Coping style may also be relevant. Active coping strategies, such as learning how to use technology and seeking help when difficulties arise, have been associated with more favourable perceptions of digital technologies among older adults [29]. In contrast, avoidance, withdrawal, or reluctance to seek help may be associated with greater perceived difficulty and lower perceived value. Social support is another relevant factor. Previous studies suggest that support from family members or others can help older adults manage technology-related difficulties [30], and may be associated with greater perceived ease of use and perceived usefulness. Social support may therefore also be indirectly associated with technology anxiety through these perceptions. Existing evidence also provides a basis for examining direct associations between these factors and technology anxiety. The present study therefore examined both their direct associations with technology anxiety and their potential indirect associations through PEOU and PU.

Previous studies have identified separate associations between individual- and social-level factors, perceptions of digital health technologies, and technology anxiety. However, how these factors are related to technology anxiety within a single theoretical framework, including both direct associations and potential indirect associations through PEOU and PU, remains insufficiently understood. The present study therefore tested an extended TAM-based model among older adults with chronic diseases.

## 2. The Study

### Aim and Hypotheses

This study aimed to examine the associations of individual-level factors (eHealth literacy, chronic disease management self-efficacy, positive coping, and negative coping) and the social-level factor (social support) with technology anxiety among older adults with chronic diseases, including their direct associations and potential indirect associations through perceived ease of use (PEOU) and perceived usefulness (PU). Given the cross-sectional design, the indirect effects were interpreted as statistical indirect associations rather than causal mediation effects.

Based on the extended TAM framework, the following hypotheses were proposed:

**H1a.**  *eHealth literacy is positively associated with PEOU and PU.*

**H1b.**  *Chronic disease management self-efficacy is positively associated with PEOU and PU.*

**H1c.**  *Positive coping is positively associated with PEOU and PU.*

**H1d.**  *Social support is positively associated with PEOU and PU.*

**H2.**  *Negative coping is negatively associated with PEOU and PU.*

**H3.**  *PEOU is positively associated with PU, and both PEOU and PU are negatively associated with technology anxiety.*

**H4a.**  *eHealth literacy shows negative indirect associations with technology anxiety through PEOU and PU.*

**H4b.**  *Chronic disease management self-efficacy shows negative indirect associations with technology anxiety through PEOU and PU.*

**H4c.**  *Positive coping shows negative indirect associations with technology anxiety through PEOU and PU.*

**H4d.**  *Social support shows negative indirect associations with technology anxiety through PEOU and PU.*

**H4e.**  *Negative coping shows positive indirect associations with technology anxiety through PEOU and PU.*

The model also included direct paths from the individual- and social-level factors to technology anxiety to allow direct and indirect associations to be estimated simultaneously rather than assuming complete mediation. The hypothesised model is shown in Figure 1.

## 3. Materials and Methods

### 3.1. Study Design

This study adopted a cross-sectional observational design and was conducted in accordance with the Strengthening the Reporting of Observational Studies in Epidemiology (STROBE) guidelines. SEM was employed to examine the relationships between latent and observed variables.

### 3.2. Study Setting and Sampling

Study preparation began in September 2025 and was limited to administrative coordination and research planning. Following ethical approval on 9 December 2025, participant screening and recruitment were conducted between December 2025 and March 2026 at the First Affiliated Hospital of Anhui Medical University, a tertiary hospital in Anhui Province, China. Participants were recruited using convenience sampling. Eligible participants were required to meet the following inclusion criteria: (1) aged 60 years or older; (2) had a documented diagnosis of hypertension, type 2 diabetes mellitus, or lung cancer in the electronic medical records of the participating hospital; (3) able to understand the questionnaire and communicate verbally; and (4) had no documented history of severe cognitive impairment or psychiatric disorders in the hospital electronic medical records that might interfere with study participation. Participants were excluded if they: (1) were currently enrolled in another clinical or interventional study; or (2) were unable to use a smartphone for healthcare-related purposes, including those without access to one. This criterion was applied because sufficient exposure to digital healthcare technologies was necessary for assessing technology anxiety, PEOU, and PU.

Sample size adequacy was assessed according to commonly used empirical criteria for SEM. A sample-to-observed-variable ratio of 10:1 to 15:1 has been suggested as an empirical guideline for SEM [31]. The main model included 16 observed variables: five composite variables (eHealth literacy, chronic disease management self-efficacy, social support, positive coping, and negative coping), four indicators of PEOU, four indicators of PU, and three dimension-level indicators of technology anxiety. Using the more conservative ratio of 15:1, approximately 240 valid cases were considered adequate. Considering a potential 20% invalid-response rate, a target sample size of approximately 300 participants was planned. The final analytic sample comprised 456 participants, exceeding this empirical guideline.

### 3.3. Measures

#### 3.3.1. Demographic and Background Characteristics

Demographic and background characteristics were collected using a researcher-developed questionnaire. Variables included gender, age, place of residence, monthly per capita household income, educational level, disease type, number of chronic diseases, disease duration, frequency of smart device or appointment system use, and willingness to use digital health technologies.

#### 3.3.2. Technology Anxiety Scale (TAS)

Technology anxiety was assessed using the Chinese version of the Technology Anxiety Scale. The original instrument was developed by Khasawneh and comprised 16 items across five dimensions [32]. The Chinese version adapted by Sun et al. contains 13 items across three dimensions: technology tension, technology fear, and privacy/security concerns [33]. Each item was rated on a 5-point Likert scale, with higher total scores indicating greater technology anxiety. In the present study, the Cronbach’s α coefficient for the total scale was 0.92.

#### 3.3.3. Perceived Ease of Use (PEOU)

Perceived ease of use was assessed using an existing four-item Chinese-language version of the PEOU measure [34]. This Chinese version had been developed in a previous Chinese study by adapting the four-item measure reported by Khan et al. [35]. In the present study, this existing Chinese version was used directly without further translation or item modification. A sample item was “Learning to use hospital self-service devices or mobile apps is easy for me.” Each item was rated on a 5-point Likert scale, with higher scores reflecting greater perceived ease of use of digital health technologies. In this study, the Cronbach’s α coefficient was 0.88.

#### 3.3.4. Perceived Usefulness (PU)

Perceived usefulness was assessed using an existing four-item Chinese-language version of the PU measure [34]. This Chinese version had been developed in a previous Chinese study by adapting the four-item measure reported by Khan et al. [35]. In the present study, this existing Chinese version was used directly without further translation or item modification. A sample item was “Using hospital self-service devices or mobile apps makes my healthcare more convenient.” Items were rated on a 5-point Likert scale, and higher scores indicated greater perceived usefulness of digital health technologies. The Cronbach’s α coefficient in this study was 0.89.

#### 3.3.5. eHealth Literacy Scale (eHEALS)

eHealth literacy was measured using the Chinese version of the eHealth Literacy Scale (eHEALS) [36], originally developed by Norman and Skinner and later adapted for use in Chinese populations [20]. The scale includes eight items and assesses individuals’ perceived ability to obtain, understand, evaluate, and apply health information from electronic sources. Higher scores indicate better eHealth literacy. In the present study, the Cronbach’s α coefficient was 0.83.

#### 3.3.6. Self-Efficacy for Managing Chronic Disease Scale (CDSES)

Chronic disease self-management self-efficacy was measured using the CDSES, developed by Lorig and colleagues at Stanford University [21]. The scale consists of six items organised into two domains: symptom management (items 1–4) and disease control (items 5–6). Each item is rated on a visual analogue scale from 1 to 10, with higher scores indicating greater self-efficacy. The scale has been widely used in research involving older adults with chronic conditions. In the present study, the Cronbach’s α coefficient was 0.84.

#### 3.3.7. Social Support Rating Scale (SSRS)

Social support was measured using the Social Support Rating Scale (SSRS), which includes 10 items covering subjective support, objective support, and support utilisation [23]. Higher scores indicate greater perceived social support. In the present study, the Cronbach’s α coefficient was 0.79.

#### 3.3.8. Simplified Coping Style Questionnaire (SCSQ)

Coping style was assessed using the Simplified Coping Style Questionnaire (SCSQ), adapted from the Ways of Coping Questionnaire and revised for use in Chinese populations [22]. The scale includes 20 items across two dimensions: positive coping and negative coping. Items are rated on a 4-point scale, with higher scores indicating greater use of the respective coping strategy. In this study, the Cronbach’s α coefficients were 0.90 for positive coping and 0.87 for negative coping, indicating good internal consistency.

### 3.4. Data Analysis

Data were analysed using IBM SPSS Statistics, version 27.0 (IBM Corp., Armonk, NY, USA), and IBM SPSS Amos, version 26.0 (IBM Corp., Armonk, NY, USA). Descriptive statistics were used to summarise participants’ demographic and background characteristics. Pearson’s correlation analyses were conducted to examine associations among the study variables. Prior to model testing, normality was assessed using skewness and kurtosis values. All variables demonstrated acceptable distributions, with skewness and kurtosis values within ±2, indicating approximate normality [37]. Multicollinearity was assessed using variance inflation factors (VIFs), all of which were below 10, indicating no evidence of multicollinearity [38]. Confirmatory factor analysis was first performed to evaluate the measurement model for the latent constructs, including technology anxiety, perceived ease of use, and perceived usefulness. Technology anxiety was specified as a latent construct represented by the scores of its three validated subdimensions: technology tension (TA1), technology fear (TA2), and privacy/security concerns (TA3). The remaining variables were entered into the SEM as observed composite variables using their total scores to examine their structural relationships with perceived ease of use, perceived usefulness, and technology anxiety, while avoiding unnecessary model complexity. Structural equation modelling was then used to test the hypothesised relationships among the study variables. The model was estimated using maximum likelihood estimation. Model fit was assessed using a combination of absolute and incremental indices. Absolute fit was evaluated with the chi-square to degrees of freedom ratio (χ^2^/df) and the root mean square error of approximation (RMSEA). Incremental fit indices included the comparative fit index (CFI), Tucker–Lewis index (TLI), and incremental fit index (IFI). Following conventional criteria, χ^2^/df values below 5 indicate acceptable fit, RMSEA values below 0.08 indicate acceptable approximation, and CFI, TLI, and IFI values above 0.90 are considered indicative of adequate model fit [37]. After the initial structural model was estimated, modification indices (MIs) were examined to improve model fit. Covariances among exogenous variables were added sequentially when suggested by the MI results and considered theoretically reasonable. Three covariance paths were added: eHealth literacy with chronic disease management self-efficacy, social support with positive coping, and positive coping with negative coping. These covariance specifications were considered conceptually plausible because eHealth literacy and chronic disease management self-efficacy both reflect individual resources relevant to health management, social support and positive coping represent related psychosocial resources, and positive and negative coping are two dimensions of the same coping framework [20,21,22,23]. The corresponding MI values and changes in model fit are reported in Section 4. Indirect effects were tested using bootstrapping with 5000 resamples and 95% bias-corrected confidence intervals. An indirect effect was considered statistically significant when the confidence interval did not include zero [39].

### 3.5. Ethical Considerations

This study was conducted in hospitals in the Hefei area of Anhui Province, China. Ethical approval was granted by the Biomedical Ethics Committee of Anhui Medical University (approval number: 81250923). Prior to completing the questionnaire, all participants were required to provide written informed consent. Any queries raised by participants were addressed verbally by the researcher. Anonymity was preserved throughout the return process.

## 4. Results

### 4.1. Sample Characteristics

A total of 470 older adults with chronic diseases were recruited for this study. After excluding incomplete or invalid responses, 456 valid questionnaires were obtained, yielding an effective response rate of 97%. As shown in Table 1, just over half of the sample were male (50.7%), and the most represented age group was 65–69 years (34.2%). The majority of participants resided in rural areas (58.8%). In terms of educational attainment, 78.7% had completed junior high school or below, and nearly half reported a monthly per capita household income below 3000 RMB (48.9%). The three chronic conditions represented were diabetes (37.9%), lung cancer (32.5%) and hypertension (29.6%). Regarding digital health technology use, 45.6% used smart devices or appointment systems occasionally, whilst 17.5% reported never using them. Just over half indicated a willingness to use digital health technologies (54.8%).

### 4.2. Descriptive and Bivariate Correlation Analyses of the Main Variables

Regarding scale scores, as presented in Table 2, the mean TAS score was 38.41 (SD = 11.80). The mean eHEALS score was 24.53 (SD = 5.94) and the mean CDSES score was 38.54 (SD = 8.44). The mean SSRS score was 35.59 (SD = 10.15), with PC and NC scoring 20.23 (SD = 8.18) and 10.77 (SD = 5.72), respectively. The mean scores for PEOU and PU were 12.09 (SD = 4.33) and 12.24 (SD = 4.33).

The bivariate correlations among study constructs are presented in Table 3. Technology anxiety was negatively correlated with eHealth literacy (*r* = −0.61, *p* < 0.01), chronic disease management self-efficacy (*r* = −0.46, *p* < 0.01), perceived ease of use (*r* = −0.61, *p* < 0.01), and perceived usefulness (*r* = −0.71, *p* < 0.01). These results indicate that higher technology anxiety was associated with lower eHealth literacy, self-efficacy, perceived ease of use, and perceived usefulness. A significant positive association was observed between technology anxiety and negative coping (*r* = 0.27, *p* < 0.01), suggesting that higher technology anxiety was linked to more negative coping, whilst social support showed a weak but significant negative correlation with technology anxiety (*r* = −0.22, *p* < 0.01), indicating that higher levels of social support were associated with slightly lower technology anxiety.

### 4.3. SEM Results

Confirmatory factor analysis was performed to evaluate the measurement model, including construct reliability and convergent validity. All the observed indicators had statistically significant standardised loadings, which ranged from 0.75 to 0.84, exceeding the commonly accepted threshold of 0.40 [40]. The Cronbach’s alpha coefficients were 0.783 for technology anxiety, 0.884 for PEOU, and 0.890 for PU. The corresponding McDonald’s omega coefficients were 0.770, 0.877, and 0.883, respectively. The composite reliability (CR) values were 0.817, 0.884, and 0.892, respectively, while the average variance extracted (AVE) values were 0.599, 0.657, and 0.673, respectively. All reliability coefficients exceeded the recommended threshold of 0.70, and all AVE values exceeded 0.50, indicating satisfactory construct reliability and convergent validity. The measurement model also showed a good fit to the data: χ^2^/df = 1.31, RMSEA = 0.03, CFI = 0.99, TLI = 0.99, IFI = 0.99. All fit indices met the stipulated criteria. No post hoc modifications were made to the model. The initial structural model showed a less satisfactory fit. Modification indices were subsequently examined, and covariance paths were added sequentially when supported by both the MI values and theoretical plausibility. The first covariance added was between eHealth literacy and chronic disease management self-efficacy (MI = 98.221), followed by the covariance between social support and positive coping (MI = 38.385). After re-estimation, the covariance between positive and negative coping was subsequently added (MI = 40.477). Model fit improved progressively across the sequential modifications. The final model demonstrated satisfactory fit: χ^2^/df = 2.647, RMSEA = 0.060, CFI = 0.961, TLI = 0.947, and IFI = 0.961. The standardised path coefficients of the structural model are presented in Figure 2 and Table 4.

H1a–H1d and H2 were supported. eHealth literacy, chronic disease management self-efficacy, positive coping, and social support were positively associated with both PEOU and PU, whereas negative coping was negatively associated with both constructs (all *p* < 0.05; Table 5). Specifically, eHealth literacy was positively associated with PEOU (β = 0.409, *p* < 0.001) and PU (β = 0.192, *p* < 0.001). H3 was also supported. PEOU was positively associated with PU (β = 0.472, *p* < 0.001), while both PEOU (β = −0.211, *p* < 0.001) and PU (β = −0.252, *p* < 0.001) were negatively associated with technology anxiety. Regarding the direct associations with technology anxiety, eHealth literacy (β = −0.310, *p* < 0.001), chronic disease management self-efficacy (β = −0.095, *p* = 0.029), and positive coping (β = −0.292, *p* < 0.001) were negatively associated with technology anxiety, whereas negative coping was positively associated with technology anxiety (β = 0.369, *p* < 0.001). Social support was not directly associated with technology anxiety (β = 0.000, *p* = 0.996). Bootstrap analyses supported H4a–H4e. For all five antecedent variables, the specific indirect effects through PEOU, through PU, and through the sequential pathway from PEOU to PU were statistically significant, with the 95% bias-corrected confidence intervals excluding zero (Table 6). eHealth literacy, chronic disease management self-efficacy, positive coping, and social support showed negative indirect associations with technology anxiety, whereas negative coping showed positive indirect associations. The indirect pathways represented 37.1% of the total association for eHealth literacy, 53.6% for self-efficacy, 100.0% for social support, 18.9% for positive coping, and 19.3% for negative coping.

## 5. Discussion

This study examined the associations of individual-level factors (eHealth literacy, chronic disease management self-efficacy, positive coping, and negative coping) and the social-level factor (social support) with technology anxiety among older adults with chronic diseases, as well as their potential indirect associations through PEOU and PU. eHealth literacy, chronic disease management self-efficacy, positive coping, and social support were positively associated with PEOU and PU, whereas negative coping was negatively associated with both. PEOU was positively associated with PU, and both PEOU and PU were negatively associated with technology anxiety. EHealth literacy, chronic disease management self-efficacy, and positive coping were directly associated with lower technology anxiety, whereas negative coping was directly associated with higher technology anxiety. Social support showed no significant direct association with technology anxiety. In addition, all five antecedent variables showed significant indirect associations with technology anxiety through PEOU, through PU, and through the sequential pathway from PEOU to PU.

The findings should be interpreted by distinguishing the original TAM component from the extensions proposed in this study. The positive association between PEOU and PU is consistent with the core relationship specified in the original TAM [24]. In contrast, the inclusion of eHealth literacy, chronic disease management self-efficacy, coping style, and social support as antecedents of PEOU and PU represents an extension of the TAM to the context of digital healthcare among older adults with chronic diseases. Similarly, positioning technology anxiety as the outcome of PEOU and PU, together with the direct associations between the antecedent factors and technology anxiety, extends beyond the original TAM. Thus, the present findings support the proposed extended framework rather than the original TAM as a whole.

Among our sample of older adults with chronic diseases, the mean TAS score approached the theoretical midpoint of the scale, suggesting a moderate level of technology anxiety in this population, broadly comparable to previous findings [41]. However, this finding should be interpreted with caution, as participants without smartphone access or the ability to use smartphones for healthcare purposes were excluded. This exclusion may have underrepresented older adults with greater digital barriers and potentially higher technology anxiety, leading to an underestimation of the overall level of technology anxiety in the broader population of older adults with chronic diseases.

eHealth literacy reflects an individual’s ability to locate, evaluate, and apply health information from digital sources [20]. Older adults with higher eHealth literacy may be more familiar with accessing and interpreting online health information and may therefore experience fewer difficulties when engaging with digital health technologies [26]. Previous studies have also linked eHealth literacy with technology anxiety. The present findings further suggest that PEOU and PU may help account for this association, indicating that how older adults with chronic diseases perceive digital technologies may be relevant to understanding the relationship between eHealth literacy and technology anxiety.

Chronic disease management self-efficacy reflects an individual’s confidence in managing the symptoms, treatment, and lifestyle adjustments associated with chronic illnesses [21]. In the present study, chronic disease management self-efficacy was positively associated with PEOU and PU and was directly associated with lower technology anxiety. Higher self-efficacy may be related to greater confidence in learning and using digital health technologies, which may contribute to more favourable perceptions of their ease of use and usefulness [42]. PEOU and PU may help explain the association between self-efficacy and technology anxiety. Dou et al. similarly found that self-efficacy was significantly associated with PEOU among chronic disease patients adopting smartphone health technology [28].

Positive coping refers to problem-focused strategies in which individuals actively seek to manage stressors, such as planning, problem-solving, and seeking constructive solutions, whereas negative coping refers to emotion-focused or avoidant strategies, such as denial, distraction, and resignation [22]. Older adults with chronic diseases who adopt more positive coping strategies may be more willing to engage with unfamiliar digital tasks and may therefore perceive digital health technologies as easier to use and more useful. In contrast, those who rely more on negative coping may be more likely to perceive these technologies as difficult or of limited value. PEOU and PU may therefore help explain the association between coping style and technology anxiety. This pattern is broadly consistent with previous evidence showing that conservative attitudes and strong attachment to existing health-management routines can act as barriers to technology adoption among older adults with chronic diseases [43].

Social support refers to tangible assistance from social networks, including family, friends and community, as well as the subjective sense of being respected, cared for and understood [23]. In the present study, social support showed no significant direct association with technology anxiety after PEOU, PU, and the other antecedent factors were considered simultaneously. However, significant indirect associations were observed through PEOU, through PU, and through the sequential PEOU-to-PU pathway. This pattern suggests that the relationship between social support and technology anxiety may be largely reflected in how older adults with chronic diseases evaluate digital health technologies, rather than in an independent direct association. Practical assistance and encouragement from family members or other social contacts may be associated with fewer perceived operational difficulties and greater perceived usefulness of digital health technologies [44]. Thus, social support may be particularly relevant to technology anxiety when it is accompanied by more favourable perceptions of the ease of use and usefulness of digital health technologies.

Taken together, these findings suggest that technology anxiety among older adults with chronic diseases is associated with both individual- and social-level factors, as well as with their perceptions of the ease of use and usefulness of digital health technologies. In practice, nurses and other healthcare professionals may consider eHealth literacy, self-efficacy, coping style, and available social support when identifying patients who may need additional assistance with digital health services. These findings may help inform the identification of older adults who are more likely to experience difficulties with digital health technologies and support the development of more targeted digital health assistance strategies.

## 6. Limitations

This study has several limitations. First, the cross-sectional design does not allow temporal or causal relationships among the study variables to be established; therefore, the observed indirect effects should be interpreted as statistical indirect associations rather than causal mediation. Second, participants were recruited through convenience sampling from a single tertiary hospital and were limited to those with hypertension, type 2 diabetes mellitus, or lung cancer, which may limit the generalisability of the findings to other populations and healthcare settings. In addition, excluding individuals without smartphone access or the ability to use smartphones for healthcare purposes may have underrepresented older adults with greater digital barriers and possibly higher technology anxiety. Third, all variables were assessed using self-reported questionnaires at the same time point, which may introduce response bias and common method bias. Fourth, the final SEM specification was partly informed by modification indices. Although the added covariance paths were theoretically plausible, the stability of the modified model should be further examined in independent samples. Finally, the study did not separately examine different types of digital healthcare technologies, and perceptions and experiences may vary across specific technologies and use contexts. Future multicentre and longitudinal studies with more diverse populations could further examine these relationships.

## 7. Conclusions

This study found that eHealth literacy, chronic disease management self-efficacy, coping style, and social support were associated with technology anxiety among older adults with chronic diseases, both directly and through perceived ease of use and perceived usefulness. Higher eHealth literacy, stronger self-efficacy, and more positive coping were associated with more favourable perceptions of ease of use and usefulness, whereas negative coping showed the opposite pattern. Perceived ease of use and perceived usefulness were both negatively associated with technology anxiety. eHealth literacy, self-efficacy, positive coping, and negative coping also showed significant direct associations with technology anxiety, while social support showed no significant direct association but remained indirectly associated through PEOU and PU. These findings suggest that cognitive evaluations of digital technologies may help explain how individual and social factors are related to technology anxiety. The findings may inform the identification of older adults with chronic diseases who need additional support when engaging with digital health technologies and provide a basis for more targeted digital health support strategies.

## Figures and Tables

**Figure 1:**
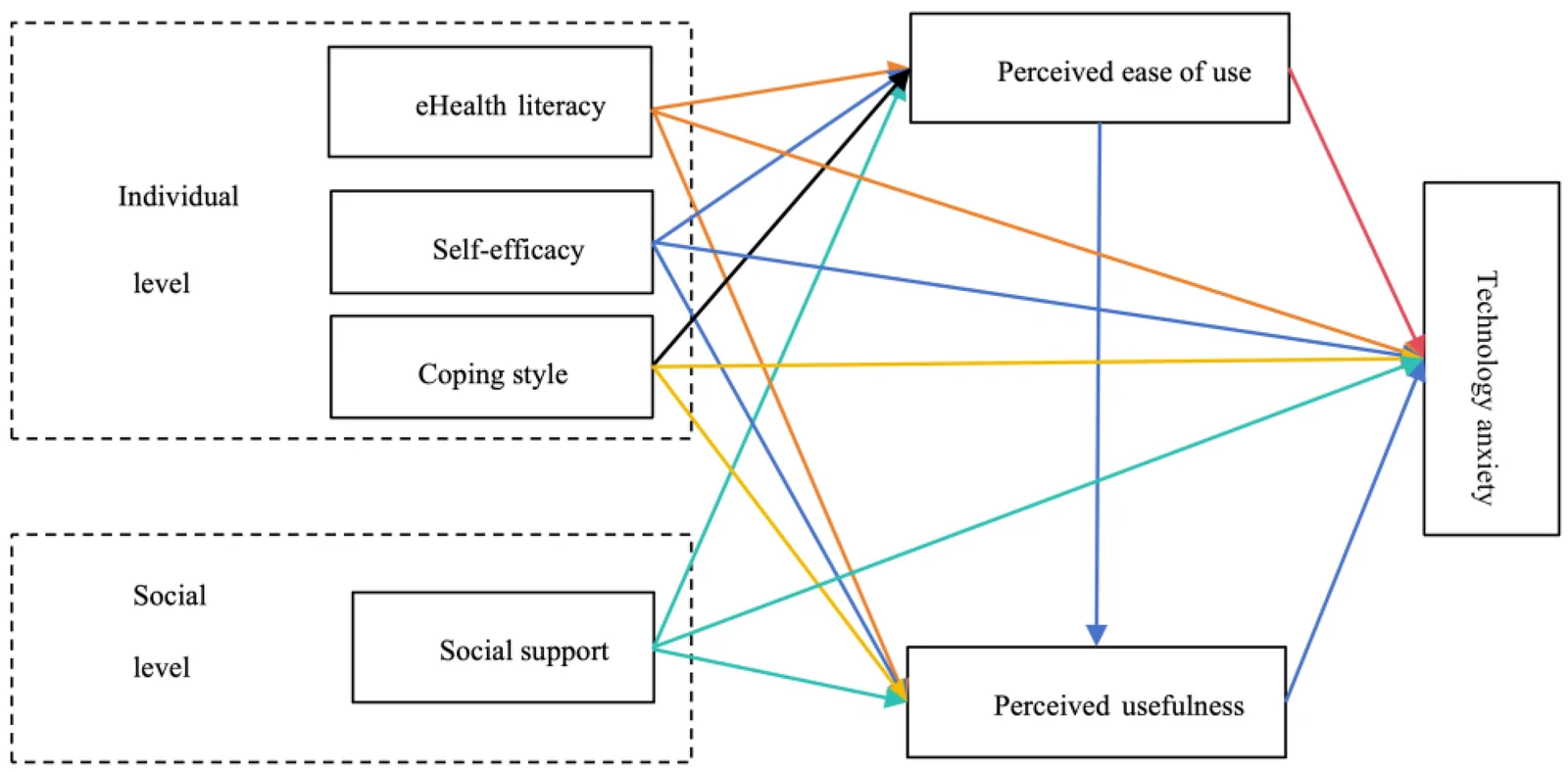
Extended Technology Acceptance Model for technology anxiety among older adults with chronic diseases. Note: Dashed boxes indicate the individual and social levels. Different arrow colors are used only for visual distinction and do not represent different types of effects.

**Figure 2:**
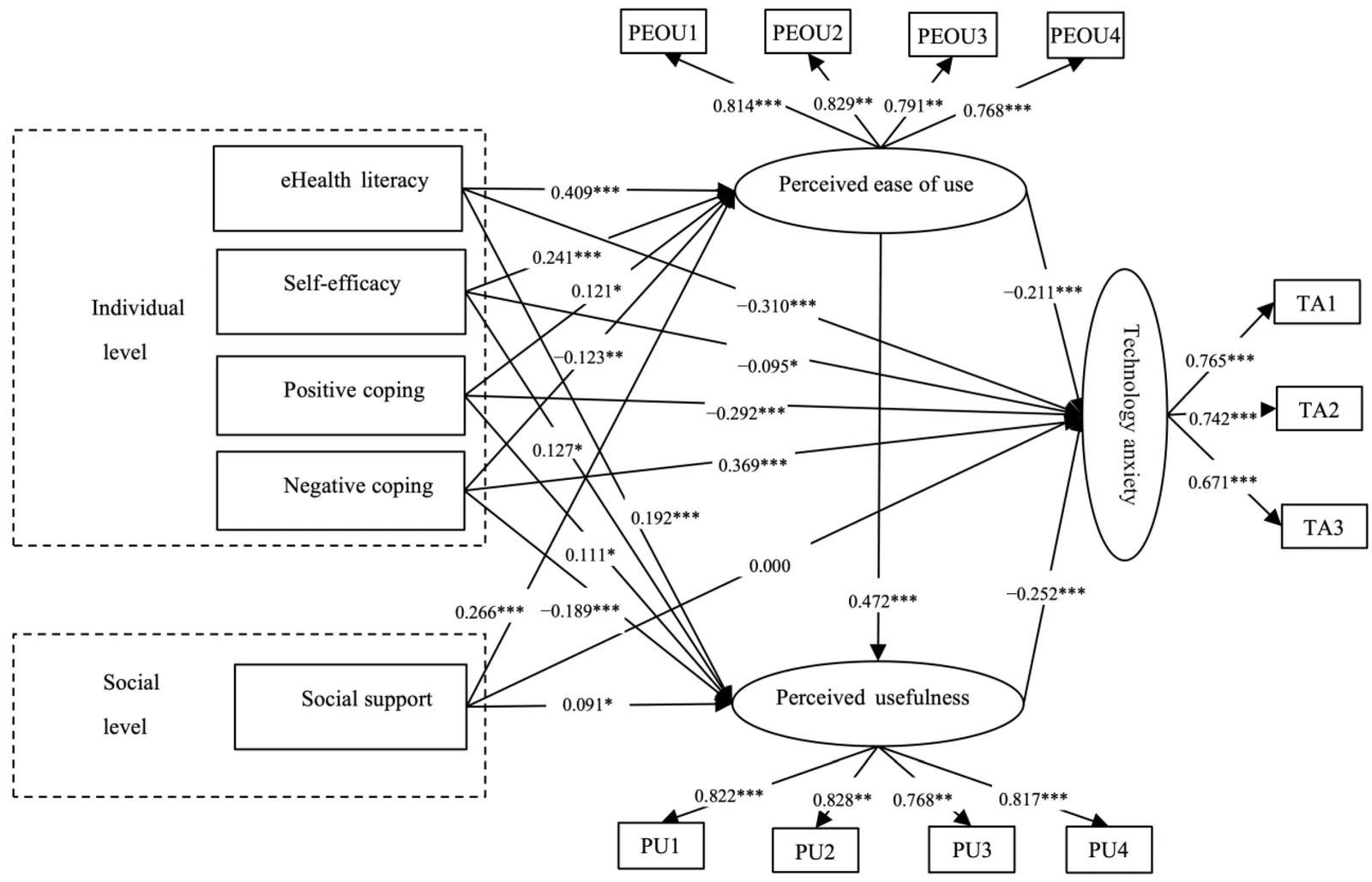
Tested model with standardised coefficients. Note: * Significance at *p* < 0.05. ** Significance at *p* < 0.01. *** Significance at *p* < 0.001. Arrows indicate the direction of the modeled associations, and dashed boxes group variables at the individual and social levels.

**Table 1:** Table of the participants (*n* = 456).

		*n*	%
Gender	Male	231	50.7
	Female	225	49.3
Age (years)	60–64	117	25.7
	65–69	156	34.2
	70–74	135	29.6
	75–79	48	10.5
Place of residence	Urban	188	41.2
	Rural	268	58.8
Monthly per capita household income	<3000 RMB	223	48.9
	3000–5000 RMB	142	31.1
	>5000 RMB	91	20.0
Education	No formal education	73	16.0
	Primary school	167	36.6
	Junior high school	119	26.1
	High school/secondary vocational	72	15.8
	College or above	25	5.5
Disease type	Hypertension	135	29.6
	Diabetes	173	37.9
	Lung cancer	148	32.5
Number of chronic diseases	1	161	35.3
	2	142	31.1
	≥3	153	33.6
Disease duration	<5 years	138	30.3
	5–10 years	151	33.1
	≥10 years	167	36.6
Smart device/appointment system use frequency	Often	168	36.8
	Occasionally	208	45.6
	Never	80	17.5
Willingness to use digital health technologies	Yes	250	54.8
	No	206	45.2

**Table 2:** Means (M) and standard deviations (SD) of TAS, eHEALS, CDSES, SSRS, PC, NC, PEOU, and PU among older adults with chronic diseases (*n* = 456).

	M	SD
eHEALS	24.53	5.94
CDSES	38.54	8.44
SSRS	35.59	10.15
PC	20.23	8.18
NC	10.77	5.72
PEOU	12.09	4.33
PU	12.24	4.33
TAS	38.41	11.80

M: mean. SD: standard deviation. TAS: Technology Anxiety Scale. eHEALS: eHealth Literacy Scale. CDSES: Self-Efficacy for Managing Chronic Disease. SSRS: Social Support Rating Scale. PC: Positive Coping. NC: Negative Coping. PEOU: Perceived Ease of Use. PU: Perceived Usefulness.

**Table 3:** Bivariate correlation coefficients of the study variables (*n* = 456).

Variables	1	2	3	4	5	6	7	8
1. eHEALS	–							
2. CDSES	0.47 **	–						
3. SSRS	0.03	0.05	–					
4. PC	0.39 **	0.42 **	0.30 **	–				
5. NC	0.01	0.13 **	−0.02	0.29 **	–			
6. PEOU	0.51 **	0.45 **	0.29 **	0.38 **	−0.05	–		
7. PU	0.50 **	0.43 **	0.26 **	0.36 **	−0.15 **	0.63 **	–	
8. TAS	−0.61 **	−0.46 **	−0.22 **	−0.44 **	0.27 **	−0.61 **	−0.71 **	–

TAS: Technology Anxiety Scale. PU: Perceived Usefulness. eHEALS: eHealth Literacy Scale. CDSES: Self-Efficacy for Managing Chronic Disease. PC: Positive Coping. NC: Negative Coping. PEOU: Perceived Ease of Use. SSRS: Social Support Rating Scale. Pearson’s r correlations are reported for associations among scale variables. ** Significance at *p* < 0.01.

**Table 4:** Comparison of fit indices across sequential structural model modifications.

Model	Model Modification	MI *	χ^2^/df	RMSEA	CFI	TLI	IFI
Original model	No exogenous covariances	-	4.683	0.09	0.91	0.881	0.911
Modified model 1	eHealth literacy ↔ chronic disease management self-efficacy	98.22	3.505	0.074	0.939	0.919	0.94
Modified model 2	social support ↔ positive coping	38.385	3.094	0.068	0.95	0.933	0.95
Final model	positive coping ↔ negative coping	40.477	2.647	0.06	0.961	0.947	0.961

Note. MI = modification index; RMSEA = root mean square error of approximation; χ^2^/df = chi-square to degrees of freedom ratio; CFI = comparative fit index; TLI = Tucker–Lewis index; IFI = incremental fit index. ↔ indicates covariance paths between variables. * The MI value refers to the modification index for the covariance path before it was added to the subsequent model.

**Table 5:** Standardised path coefficients of the structural equation model.

Path	Estimate	*p*	Results
EHEALS → PEOU	0.409	<0.001	Supported
EHEALS → PU	0.192	<0.001	Supported
CDSES → PEOU	0.241	<0.001	Supported
CDSES → PU	0.127	0.005	Supported
PC → PEOU	0.121	0.015	Supported
PC → PU	0.111	0.017	Supported
NC → PEOU	−0.123	0.003	Supported
NC → PU	−0.189	<0.001	Supported
SSRS → PEOU	0.266	<0.001	Supported
SSRS → PU	0.091	0.029	Supported
PEOU → PU	0.472	<0.001	Supported
PEOU → TAS	−0.211	<0.001	Supported
PU → TAS	−0.252	<0.001	Supported
EHEALS → TAS	−0.310	<0.001	Supported
CDSES → TAS	−0.095	0.029	Supported
SSRS → TAS	0	0.996	Not supported
PC → TAS	−0.292	<0.001	Supported
NC → TAS	0.369	<0.001	Supported

→ indicates a direct path between variables.

**Table 6:** Standardized Specific Indirect, Total Indirect, and Total Effects With 95% Bias-Corrected Bootstrap Confidence Intervals.

Panel A. Specific Indirect Effects						
Path		Estimate	SE	Lower	Upper	*p*
eHealth literacy → PEOU → Technology anxiety		−0.086	0.028	−0.147	−0.039	<0.001
eHealth literacy → PEOU → PU → Technology anxiety		−0.049	0.015	−0.084	−0.025	<0.001
Self-efficacy → PEOU → Technology anxiety		−0.051	0.018	−0.092	−0.021	<0.001
Self-efficacy → PEOU → PU → Technology anxiety		−0.029	0.011	−0.056	−0.013	<0.001
Social support → PEOU → Technology anxiety		−0.056	0.017	−0.096	−0.027	<0.001
Social support → PEOU → PU → Technology anxiety		−0.032	0.011	−0.058	−0.015	<0.001
Positive coping → PEOU → Technology anxiety		−0.025	0.014	−0.062	−0.005	0.01
Positive coping → PEOU → PU → Technology anxiety		−0.014	0.007	−0.033	−0.003	0.008
Negative coping → PEOU → Technology anxiety		0.026	0.012	0.008	0.057	0.003
Negative coping → PEOU → PU → Technology anxiety		0.015	0.006	0.005	0.031	0.002
eHealth literacy → PU → Technology anxiety		−0.048	0.021	−0.102	−0.017	<0.001
Self-efficacy → PU → Technology anxiety		−0.032	0.015	−0.070	−0.009	0.007
Social support → PU → Technology anxiety		−0.023	0.012	−0.054	−0.003	0.023
Positive coping → PU → Technology anxiety		−0.028	0.016	−0.070	−0.004	0.018
Negative coping → PU → Technology anxiety		0.048	0.018	0.019	0.091	<0.001
**Panel B. Total Indirect and Total Effects on Technology Anxiety**						
**Antecedent Variable → Technology Anxiety**	**Effect Type**	**Estimate**	**SE**	**Lower**	**Upper**	***p***
eHealth literacy → TAS	Direct effect	−0.310	0.056	−0.419	−0.202	<0.001
	Total indirect effect	−0.183	0.037	−0.263	−0.121	<0.001
	Total effect	−0.493	0.045	−0.581	−0.402	<0.001
Self-efficacy → TAS	Direct effect	−0.095	0.044	−0.181	−0.008	0.037
	Total indirect effect	−0.111	0.024	−0.164	−0.069	<0.001
	Total effect	−0.207	0.045	−0.293	−0.115	<0.001
Social support → TAS	Direct effect	0	0.039	−0.078	0.073	0.993
	Total indirect effect	−0.111	0.022	−0.161	−0.073	<0.001
	Total effect	−0.111	0.04	−0.192	−0.033	0.006
Positive coping → TAS	Direct effect	−0.292	0.051	−0.397	−0.196	<0.001
	Total indirect effect	−0.068	0.022	−0.117	−0.028	<0.001
	Total effect	−0.360	0.053	−0.465	−0.255	<0.001
Negative coping → TAS	Direct effect	0.369	0.043	0.289	0.457	<0.001
	Total indirect effect	0.088	0.022	0.049	0.135	<0.001
	Total effect	0.457	0.041	0.379	0.539	<0.001

Note. Estimates are standardized effects. SE = bootstrap standard error; PEOU = perceived ease of use; PU = perceived usefulness. → indicates a direct path between variables. Lower and upper bounds represent 95% bias-corrected bootstrap confidence intervals. An indirect effect was considered statistically significant when the 95% confidence interval did not include zero. Given the cross-sectional design, indirect effects should be interpreted as statistical indirect associations rather than causal mediation effects.

## Data Availability

The data presented in this study are available from the corresponding author upon reasonable request. Due to privacy and ethical restrictions, the dataset cannot be publicly shared.
